# Correction: A simple and reliable method for claustrum localization across age in mice

**DOI:** 10.1186/s13041-025-01171-4

**Published:** 2025-01-23

**Authors:** Tarek Shaker, Gwyneth J. Dagpa, Vanessa Cattaud, Brian A. Marriott, Mariam Sultan, Mohammed Almokdad, Jesse Jackson

**Affiliations:** 1 Department of Physiology, University of Alberta, 7‐22 Medical Sciences Building, Edmonton, T6G 2H7 AB Canada; 2 Neuroscience and Mental Health Institute, University of Alberta, Edmonton, AB Canada

**Correction: Molecular Brain (2024) 17: 10** 10.1186/s13041-024-01082-w

Following publication of the original article [1], the authors identified in the Supplementary Material file 1 that some of the images did not appear. Specifically the venn diagrams in Supplementary Figs. 4 and 5.

The incorrect (Supplementary material 2) and correct Supplementary Material file 1 (Supplementary Material file 1) are included in this publication.

The Supplementary Material file 1 has been updated and the original article [1] has been corrected.

## Supplementary Information

Supplementary Material 1.

Supplementary Material 2.

## References

[1] Shaker T, Dagpa GJ, Cattaud V, et al. A simple and reliable method for claustrum localization across age in mice. Mol Brain. 2024;17:10. 10.1186/s13041-024-01082-w. 38368400 10.1186/s13041-024-01082-w PMC10874566

