# Supplementary material for: A simple and reliable method for claustrum localization across age in mice
Source: Mol Brain. 2024 Feb 17;17:10. doi: 10.1186/s13041-024-01082-w (PMC10874566; doi:10.1186/s13041-024-01082-w)
Supplement: Supplementary file 2 — Additional file 2: Table S1. Individualized marker quantification and colocalization in the claustrum (CLA), primary motor cortex (MOp), and somatosensory cortex (SSC) for each mouse at different postnatal ages. Values represent mean cell count per slice. For the claustrum, the mean was calculated by averaging data across the anterior, middle, and posterior subdivisions. The first column reflects mouse age, where P(x) indicates postnatal day when tissue was collected. For each age, mice were derived from ≥ 2 litters. F female, M Male. Table S2. Quantification and one-way ANOVA analysis of marker colocalization with GFP+ cells in different claustrocortical anteroposterior subdivisions of the claustrum (CLA), following retrograde tracing from different cortical regions. ACC: anterior cingulate cortex, MOp: primary motor cortex, LEC: lateral entorhinal cortex, RSC: retrosplenial cortex, std dev: standard deviation. Mean ± std dev values represent the percentage of marker colocalization with GFP+ cells relative to total GFP+ cells. Table S3. Individualized marker quantification and colocalization with claustrum cells that express Tdtomato (TdT) for each mouse at different postnatal stages. AAVretro-CAG-TdT was injected into the anterior cingulate. The values represent mean cell count within coronal slices, averaged across the anterior, middle, and posterior claustrum for individual mice. The first column reflects mouse age, where P(x) indicates postnatal day when tissue was collected. For each age, mice were derived from ≥ 2 litters. F female, M Male. Datasets 1 and 2 were either derived from adjacent slices or different mice. Table S4. Individualized marker quantification and colocalization with claustrum cells that express cFos for each mouse in the naive and open field (OF) groups. Numbers represent mean cell count per slice, averaged across the anterior, middle, and posterior claustrum. The first column reflects mouse age, where P(x) indicates the postnatal day when [file 13041_2024_1082_MOESM2_ESM.docx]

**Table S1:**

Individualized marker quantification and colocalization in the claustrum (CLA), primary motor cortex (MOp), and somatosensory cortex (SSC) for each mouse at different postnatal ages. Values represent mean cell count per slice. For the claustrum, the mean was calculated by averaging data across the anterior, middle, and posterior subdivisions. The first column reflects mouse age, where P(x) indicates postnatal day when tissue was collected. For each age, mice were derived from ≥ 2 litters. F: female, M: Male.

| Mouse ID | Sex | Region | Nurr1+ | Tle4+ | Nurr1+/Tle4+ |
| --- | --- | --- | --- | --- | --- |
| P14-1 | F | CLA | 328.6 | 113.8 | 5.0 |
|  |  | MOp | 38.5 | 159.8 | 38.3 |
|  |  | SSC | 56.0 | 164.8 | 54.5 |
| P14-2 | F | CLA | 258.7 | 118.2 | 3.0 |
|  |  | MOp | N/A | N/A | N/A |
|  |  | SSC | N/A | N/A | N/A |
| P14-3 | F | CLA | 110.5 | 58.0 | 0.5 |
|  |  | MOp | N/A | N/A | N/A |
|  |  | SSC | N/A | N/A | N/A |
| P14-5 | M | CLA | 288.0 | 163.3 | 2.8 |
|  |  | MOp | 33.5 | 113.8 | 32.8 |
|  |  | SSC | 58.5 | 184.0 | 58.5 |
| P14-6 | M | CLA | 230.0 | 104.7 | 0.5 |
|  |  | MOp | 53.5 | 154.8 | 51.0 |
|  |  | SSC | 57.0 | 171.3 | 55.5 |
| P14-7 | M | CLA | 207.7 | 83.7 | 0.7 |
|  |  | MOp | N/A | N/A | N/A |
|  |  | SSC | N/A | N/A | N/A |
| P75-1 | F | CLA | 163.4 | 69.6 | 0.6 |
|  |  | MOp | 42.3 | 133.8 | 41.8 |
|  |  | SSC | 69.0 | 158.5 | 68.3 |
| P75-2 | F | CLA | 177.8 | 98.0 | 0.6 |
|  |  | MOp | 54.3 | 175.5 | 54.3 |
|  |  | SSC | 77.8 | 191.3 | 75.5 |
| P75-3 | M | CLA | 210.3 | 78.0 | 2.7 |
|  |  | MOp | 32.5 | 106.3 | 32.5 |
|  |  | SSC | 57.5 | 134.5 | 56.3 |
| P75-4 | M | CLA | 174.0 | 88.0 | 5.3 |
|  |  | MOp | 17.8 | 98.3 | 17.8 |
|  |  | SSC | 36.3 | 101.0 | 33.5 |

**Table S2:**

Quantification and one-way ANOVA analysis of marker colocalization with GFP^+^ cells in different claustrocortical anteroposterior subdivisions of the claustrum (CLA), following retrograde tracing from different cortical regions. ACC: anterior cingulate cortex, MOp: primary motor cortex, LEC: lateral entorhinal cortex, RSC: retrosplenial cortex, std dev: standard deviation. Mean ± std dev values represent the percentage of marker colocalization with GFP^+^ cells relative to total GFP^+^ cells.

| Colocalization measurement | CLA subdivision | Cortical injection region (number of mice) | Mean ± std dev | F-value | p-value |
| --- | --- | --- | --- | --- | --- |
| Nurr1^+^GFP^+^ /GFP^+^ | Anterior | RSC (6) | 92.8 ± 2.0% | F(_3,15_) = 22.34 | 8.7 x 10^-6^ |
|  |  | ACC (5) | 75.0 ± 1.6% |  |  |
|  |  | MOp (4) | 59.9 ± 11.7% |  |  |
|  |  | LEC (4) | 66.5 ± 9.1% |  |  |
|  | Middle | RSC (6) | 94.6 ± 2.6% | F(_3,15_) = 19.82 | 1.8 x 10^-5^ |
|  |  | ACC (5) | 77.1 ± 3.9% |  |  |
|  |  | MOp (4) | 68.6 ± 5.3% |  |  |
|  |  | LEC (4) | 76.0 ± 10.0% |  |  |
|  | Posterior | RSC (5) | 87.8 ± 5.5%% | F(_3,14_) = 15.30 | 1.1 x 10^-4^ |
|  |  | ACC (5) | 76.2 ± 7.6% |  |  |
|  |  | MOp (4) | 50.6 ± 13.1% |  |  |
|  |  | LEC (4) | 77.4 ± 6.0% |  |  |
| Nr2f2^+^GFP^+^ /GFP^+^ | Anterior | RSC (5) | 83.5 ± 6.9% | F(_3,12_) = 24.17 | 3.8 x 10^-5^ |
|  |  | ACC (3) | 58.4 ± 0.5% |  |  |
|  |  | MOp (4) | 49.3 ± 6.7% |  |  |
|  |  | LEC (4) | 33.8 ± 13.7% |  |  |
|  | Middle | RSC (5) | 88.0 ± 9.2% | F(_3,12_) = 25.79 | 1.6 x 10^-5^ |
|  |  | ACC (3) | 53.0 ± 11.4% |  |  |
|  |  | MOp (4) | 48.3 ± 10.9% |  |  |
|  |  | LEC (4) | 26.6 ± 11.8% |  |  |
|  | Posterior | RSC (5) | 81.4 ± 8.9% | F(_3,12_) = 18.62 | 8.3 x 10^-5^ |
|  |  | ACC (3) | 53.3 ± 10.6% |  |  |
|  |  | MOp (4) | 44.6 ± 16.5% |  |  |
|  |  | LEC (4) | 24.1 ± 9.8% |  |  |
| Tle4^+^GFP^+^ /GFP^+^ | Anterior | RSC (5) | 1.2 ± 2.0% | F(_3,12_) = 2.79 | 0.090 |
|  |  | ACC (3) | 2.7 ± 0.2% |  |  |
|  |  | MOp (4) | 3.8 ± 1.7% |  |  |
|  |  | LEC (4) | 1.0 ± 1.1% |  |  |
|  | Middle | RSC (5) | 0.4 ± 0.8% | F(_3,12_) = 1.88 | 0.187 |
|  |  | ACC (3) | 2.0 ± 2.1% |  |  |
|  |  | MOp (4) | 2.6 ± 1.9% |  |  |
|  |  | LEC (4) | 1.4 ± 1.2% |  |  |
|  | Posterior | RSC (5) | 1.0 ± 1.4% | F(_3,12_) = 3.19 | 0.062 |
|  |  | ACC (3) | 2.8 ± 1.4% |  |  |
|  |  | MOp (4) | 4.9 ± 2.8% |  |  |
|  |  | LEC (4) | 2.3 ± 1.7% |  |  |

**Table S3:**

Individualized marker quantification and colocalization with claustrum cells that express Tdtomato (TdT) for each mouse at different postnatal stages. AAVretro-CAG-TdT was injected into the anterior cingulate. The values represent mean cell count within coronal slices, averaged across the anterior, middle, and posterior claustrum for individual mice. The first column reflects mouse age, where P(x) indicates postnatal day when tissue was collected. For each age, mice were derived from ≥ 2 litters. F: female, M: Male. Datasets 1 and 2 were either derived from adjacent slices or different mice.

| Mouse ID | Sex | TdT+  (dataset 1) | Nurr1+  (dataset 1) | TdT+ /Nurr1+  (dataset 1) | Tle4+  (dataset1) | TdT+/Tle4+  (dataset 1) | TdT+  (dataset 2) | Nr2f2+  (dataset 2) | TdT+/Nr2f2+  (dataset 2) |
| --- | --- | --- | --- | --- | --- | --- | --- | --- | --- |
| P7-1 | F | 40.8 | 320.0 | 27.6 | 125.0 | 1.6 | 41.0 | 290.2 | 23.2 |
| P7-2 | F | 36.3 | 309.2 | 30.2 | 96.0 | 1.5 | N/A | N/A | N/A |
| P7-3 | M | 13.4 | 213.0 | 10.6 | 47.0 | 0.2 | N/A | N/A | N/A |
| P7-4 | M | 11.5 | 165.5 | 9.8 | 42.3 | 0.5 | N/A | N/A | N/A |
| P7-5 | M | 13.4 | 358.2 | 11.8 | 79.0 | 0.4 | 13.5 | 149.5 | 8.8 |
| P7-6 | M | N/A | N/A | N/A | N/A | N/A | 15.0 | 204.5 | 11.3 |
| P7-8 | M | N/A | N/A | N/A | N/A | N/A | 31.6 | 324.2 | 20.2 |
| P7-9 | M | N/A | N/A | N/A | N/A | N/A | 45.8 | 240.4 | 30.4 |
| P14-1 | F | 30.0 | 246.8 | 23.6 | 38.4 | 0.6 | 24.0 | 197.6 | 19.8 |
| P14-2 | F | 28.3 | 192.3 | 25.3 | 43.7 | 1.2 | 23.7 | 160.7 | 20.8 |
| P14-3 | F | 24.0 | 208.0 | 19.3 | 54.8 | 0.7 | 19.3 | 130.5 | 17.7 |
| P14-4 | F | N/A | N/A | N/A | N/A | N/A | 15.0 | 171.2 | 13.2 |
| P14-5 | M | 20.8 | 253.6 | 17.0 | 106.0 | 0.8 | N/A | N/A | N/A |
| P14-6 | M | 21.2 | 213.7 | 19.2 | 47.5 | 0.5 | N/A | N/A | N/A |
| P14-7 | M | 34.8 | 225.7 | 28.8 | 67.8 | 0.7 | N/A | N/A | N/A |
| P21-1 | F | 31.0 | 246.8 | 28.2 | 124.2 | 0.8 | N/A | N/A | N/A |
| P21-2 | F | 67.0 | 158.5 | 44.0 | 41.5 | 2.5 | 58.8 | 154.5 | 26.8 |
| P21-3 | F | 44.7 | 195.8 | 37.8 | 51.3 | 1.2 | 52.8 | 159.7 | 37.7 |
| P21-4 | F | 42.8 | 194.8 | 38.5 | 56.3 | 0.8 | 46.7 | 142.8 | 34.7 |
| P21-5 | F | 29.0 | 162.2 | 25.0 | 52.5 | 0.5 | 27.5 | 158.5 | 22.7 |
| P21-6 | M | 60.2 | 179.2 | 54.8 | 65.8 | 2.7 | 62.2 | 150.8 | 57.5 |
| P21-7 | M | 23.0 | 136.7 | 17.7 | 61.2 | 0.8 | 13.8 | 76.0 | 9.2 |
| P55-1 | F | 215.5 | 54.5 | 50.0 | 62.3 | 1.3 | 43.2 | 136.3 | 28.2 |
| P55-2 | F | 226.8 | 60.2 | 48.2 | 197.4 | 1.0 | N/A | N/A | N/A |
| P55-3 | F | 217.2 | 66.0 | 56.2 | 152.8 | 1.4 | N/A | N/A | N/A |
| P55-4 | M | 267.0 | 66.0 | 55.3 | 121.2 | 2.2 | 54.0 | 171.7 | 41.7 |
| P55-5 | M | 267.3 | 66.8 | 57.0 | 131.2 | 2.8 | 49.7 | 156.7 | 35.7 |

**Table S4:**

Individualized marker quantification and colocalization with claustrum cells that express cFos for each mouse in the naive and open field (OF) groups. Numbers represent mean cell count per slice, averaged across the anterior, middle, and posterior claustrum. The first column reflects mouse age, where P(x) indicates the postnatal day when tissue was collected. F: female, M: Male.

| Mouse ID | Sex | Group | cFos | Nurr1 | cFos+/Nurr1+ | Tle4 | cFos+/Tle4+ |
| --- | --- | --- | --- | --- | --- | --- | --- |
| P90-1 | F | Naive | 13.5 | 113.7 | 7.7 | 137.2 | 0.7 |
| P90-2 | F | Naive | 21.3 | 444.5 | 14.2 | 74.8 | 0.7 |
| P90-3 | M | Naive | 9.2 | 181.2 | 5.0 | 123.2 | 0.0 |
| P90-4 | M | Naive | 10.0 | 117.0 | 5.3 | 103.2 | 0.3 |
| P90-5 | M | Naive | 0.7 | 124.7 | 0.2 | 114.2 | 0.0 |
| P90-6 | F | OF | 66.0 | 213.2 | 39.0 | 113.3 | 4.2 |
| P90-7 | F | OF | 59.3 | 181.0 | 37.0 | 94.7 | 2.8 |
| P90-8 | F | OF | 51.5 | 228.5 | 28.7 | 171.3 | 1.5 |
| P90-9 | F | OF | 60.2 | 206.8 | 32.0 | 261.0 | 0.3 |
| P90-10 | F | OF | 104.7 | 273.7 | 67.5 | 178.0 | 7.8 |
| P90-11 | F | OF | 63.3 | 211.0 | 48.7 | 149.3 | 3.2 |
| P90-12 | M | OF | 70.3 | 211.0 | 53.0 | 117.7 | 4.7 |
| P90-13 | M | OF | 68.0 | 222.3 | 44.3 | 137.3 | 5.7 |
| P90-14 | M | OF | 49.8 | 177.2 | 24.2 | 84.7 | 2.7 |
| P90-15 | M | OF | 64.7 | 200.3 | 35.0 | 129.0 | 6.8 |
