# Supplementary material for: Correction: A simple and reliable method for claustrum localization across age in mice
Source: Mol Brain. 2025 Jan 23;18:5. doi: 10.1186/s13041-025-01171-4 (PMC11760672; doi:10.1186/s13041-025-01171-4)
Supplement: Supplementary file 2 — Supplementary Material 2. [file 13041_2025_1171_MOESM2_ESM.pdf]

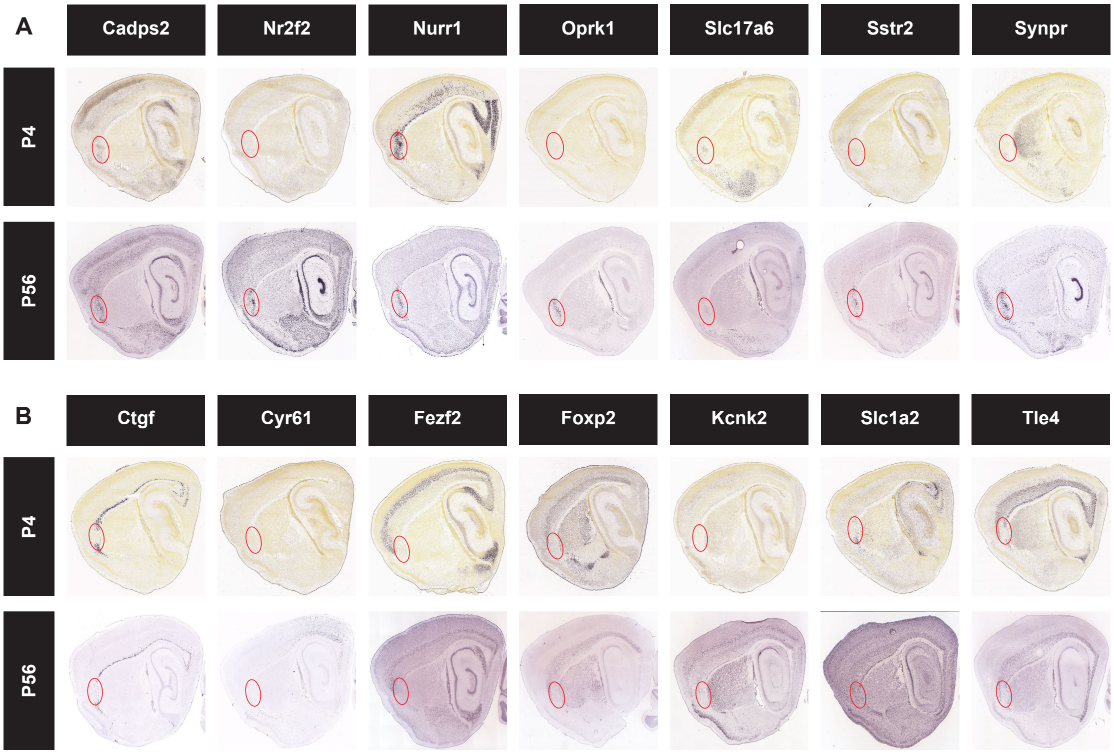

Supplementary Figure 1: Selection of candidate marker genes that are absent in claustrum cells and at the same time enriched in other nearby cells. A-B: Chromogenic *in situ* hybridization images at P4 (top) and P56 (bottom) of claustrum-enriched genes **(A)** and cortical-enriched genes **(B)** in the claustrum region (red ellipse). Candidate genes were selected based on earlier transcriptomic data showing differential expression of these genes in claustrum cells versus cortical cells (23). Images were obtained from Allen mouse brain in situ hybridization database (http://developingmouse.brain-map.org).

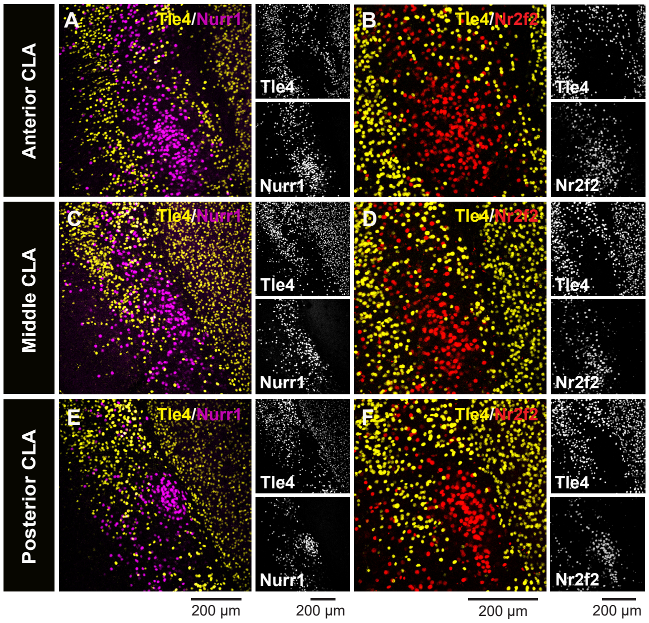

Supplementary Figure 2: Expression of the claustrum-enriched markers Nurr1 and Nr2f2 relative to the expression of Tle4 across the anteroposterior axis of the claustrum. A-F: Representative images of the anterior **(A, B)**, middle **(C, D)** and posterior **(E, F)** claustrum showing co-labeling of Nurr1 **(A, C, E)** and Nr2f2 **(B, D, F)** with Tle4 (left, merged imaging channels; right, single channel images).

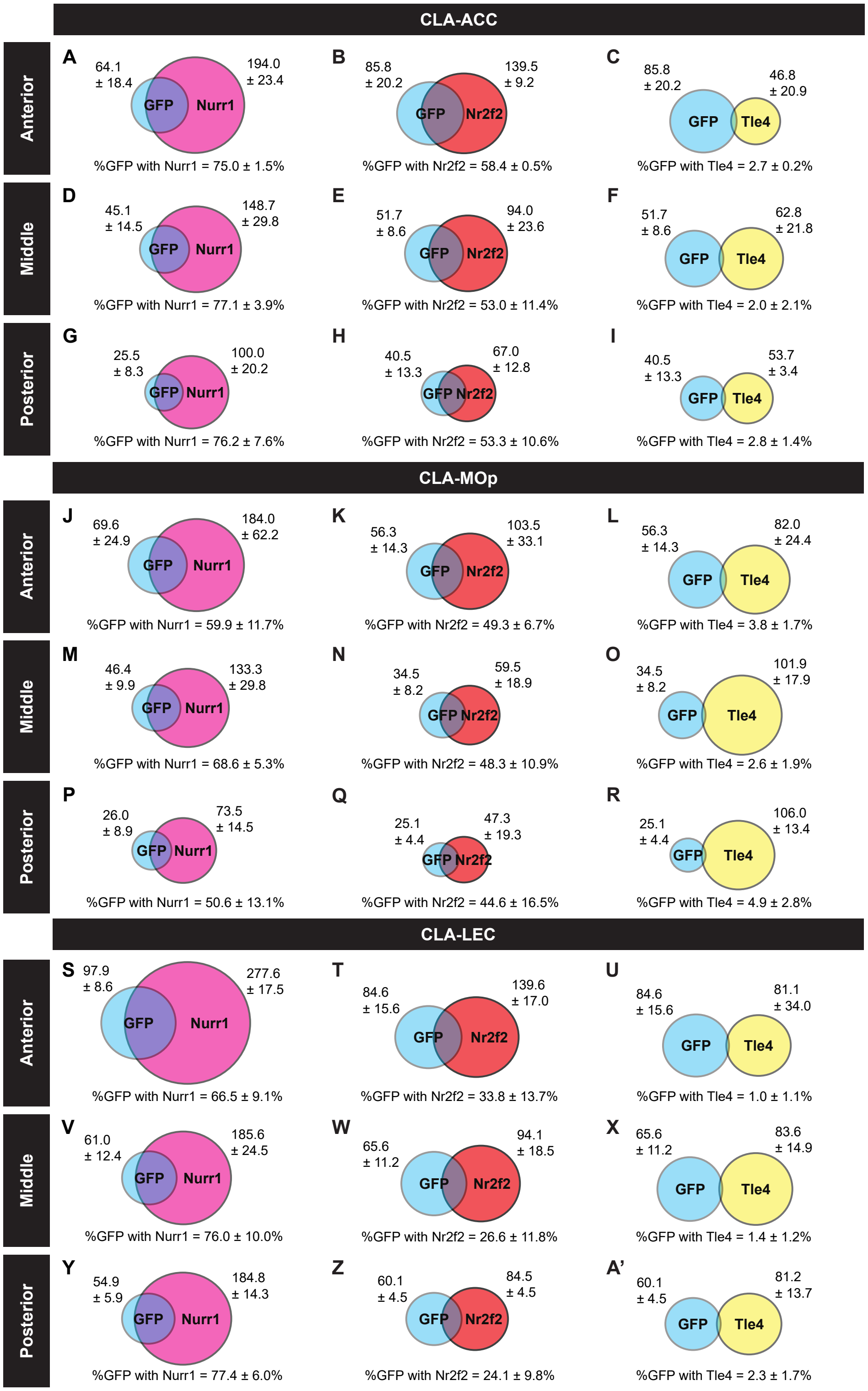

Supplementary Figure 3: The extent of colocalization between claustrum projection neurons with Nurr1, Nr2f2 and Tle4 varies based on cortical target region and cell position along the anteroposterior axis. A-I: Venn diagrams showing the number of cells expressing GFP with Nurr1 **(A, D, G)**, Nr2f2 **(B, E, H)**, and Tle4 **(C, F, I)** in the anterior **(A, B, C)**, middle **(D, E, F)** and posterior **(G, H, I)** claustrum (CLA) for neurons projecting to the anterior cingulate cortex (ACC) (n = 5 mice, all male). **J-R** Same as **A-I** but for claustrum neurons projecting to the primary motor cortex (MOp) (n = 4 mice, all male). **S-A’:** Same as **A-I**, but for claustrum neurons projecting to the lateral entorhinal cortex (LEC) (n = 4 mice, all male). Values represent mean ± standard deviation.

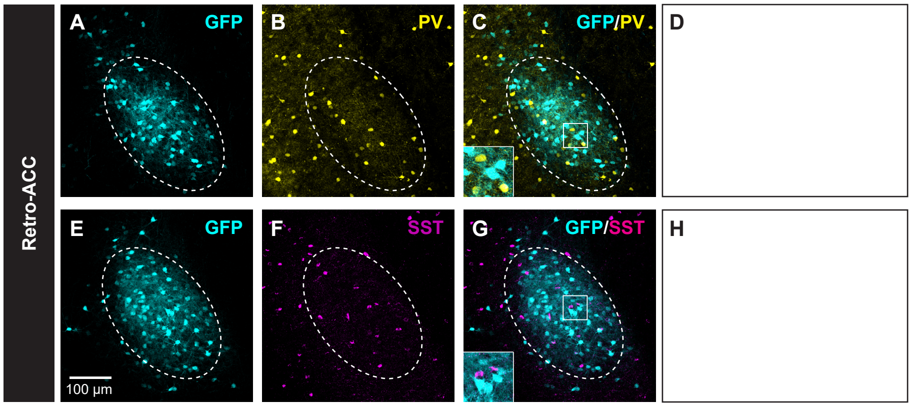

Supplementary Figure 4: Lack of colocalization between claustrum projection neurons and inhibitory markers. A-C: Representative images of the anterior claustrum (CLA, dashed ellipses) showing retrograde GFP labeling **(A)**, PV expression **(B)**, merged A and B channels **(C)** following AAV injection into the anterior cingulate cortex (Retro-ACC). **D:** Venn diagram representing the mean number of cells expressing GFP (cyan) and PV (yellow), along with the colocalization of GFP with PV relative to the total number of GFP-expressing cells. Values shown are the average cell counts from the anterior, middle and posterior planes of the CLA. **E-H:** Same as A-D, respectively, but for SST (magenta). Insets in C, G (bottom-left) are 2× fold magnifications of areas in white boxes. Values in D, H represent mean ± standard deviation (n = 5 mice, all male).

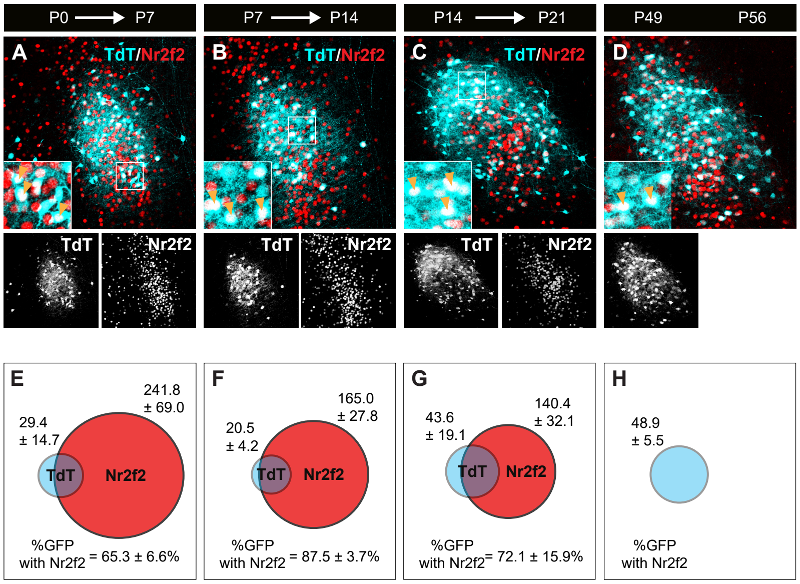

Supplementary Figure 5: Nr2f2 displays high colocalization with ACC-projecting claustrum cells consistently throughout postnatal development. (A-H) Same experimental design as in Figure 5. **A-D**: Representative images showing colocalization of TdTomato (TdT) labeling with Nr2f2 in the anterior claustrum at P7 **(A)**, P14 **(B)**, P21 **(C)** and P56 **(D)** (left, merged imaging channels; right, single channel images). Insets (bottom-left) are 2.5× fold magnifications of areas in white boxes. Orange arrowheads indicate examples of cell colocalization. **E-H:** Venn diagrams representing the mean number of cells expressing TdT (cyan) and Nr2f2 (red), along with the colocalization of TdT with Nr2f2 relative to the total number of TdT-expressing cells, at P7 **(E)**, at P14 **(F)**, at P21 **(G)** and at P56 **(H)**. Values shown represent mean ± standard deviation, which were calculated by averaging cell counts from the anterior, middle and posterior planes of the CLA (P7: n = 5 mice, P14: n = 4 mice, P21: n = 6 mice, P56: n = 3 mice) (see **Supplementary Table 3** for details on mouse sex).

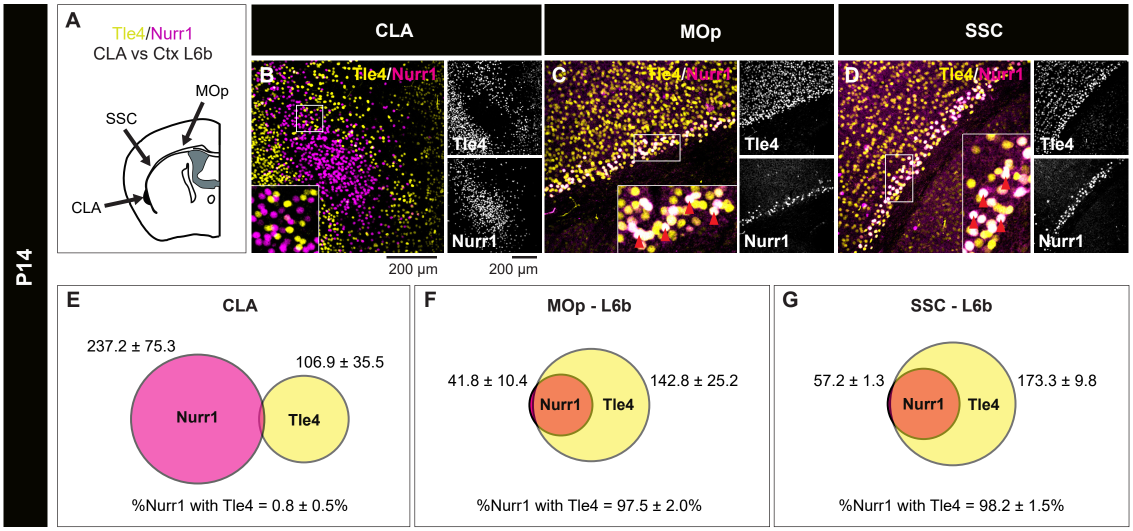

Supplementary Figure 6: Colocalization of Nurr1 with Tle4 in the claustrum is different from that in the cortex at P14. A: Schematic of the experimental design at P14 comparing Nurr1 and Tle4 colocalization in the claustrum (CLA) to cortical layer 6b (Ctx L6b) in the primary motor cortex (MOp) and the somatosensory cortex (SSC). **B-D:** Representative images showing Nurr1 and Tle4 expression in the CLA **(B)**, the MOp **(C)** and the SSC **(D)** (left, merged imaging channels; right, single channel images). Insets are 2.5× fold magnifications of areas in white boxes. Red arrowheads indicate examples of cell colocalization. **E-G:** Venn diagrams representing the mean number of cells expressing Nurr1 (magenta) and Tle4 (yellow), along with the colocalization of Nurr1 with Tle4 relative to the total number of Nurr1-expressing cells in the CLA **(E)** (n = 6 mice, 3 male and 3 female), and Ctx L6b in the MOp **(F)** and in the SSC **(G)** (n = 3 mice, 2 male and 1 female) (See **Supplementary Table 1** for details on mouse sex). Insets in B, C, D are 2.5× fold magnifications of areas in white boxes. Values shown represent mean ± standard deviation. The mean for the CLA was calculated by averaging cell counts from the anterior, middle and posterior planes of the CLA region. The mean for Ctx L6b in MOp and SSC was calculated by averaging cell counts from slices on the same plane as the anterior, middle and posterior regions of the CLA.
